# “Going with the flow” in modeling fibrinolysis

**DOI:** 10.3389/fcvm.2022.1054541

**Published:** 2022-12-02

**Authors:** Claire S. Whyte, Nicola J. Mutch

**Affiliations:** Aberdeen Cardiovascular and Diabetes Centre, Institute of Medical Sciences, School of Medicine, Medical Sciences and Nutrition, University of Aberdeen, Aberdeen, United Kingdom

**Keywords:** fibrinolysis, plasminogen, shear, fibrin, platelets, thrombus, flow

## Abstract

The formation of thrombi is shaped by intravascular shear stress, influencing both fibrin architecture and the cellular composition which has downstream implications in terms of stability against mechanical and fibrinolytic forces. There have been many advancements in the development of models that incorporate flow rates akin to those found *in vivo*. Both thrombus formation and breakdown are simultaneous processes, the balance of which dictates the size, persistence and resolution of thrombi. Therefore, there is a requirement to have models which mimic the physiological shear experienced within the vasculature which in turn influences the fibrinolytic degradation of the thrombus. Here, we discuss various assays for fibrinolysis and importantly the development of novel models that incorporate physiological shear rates. These models are essential tools to untangle the molecular and cellular processes which govern fibrinolysis and can recreate the conditions within normal and diseased vessels to determine how these processes become perturbed in a pathophysiological setting. They also have utility to assess novel drug targets and antithrombotic drugs that influence thrombus stability.

## Introduction

Fibrinolysis is the process by which fibrin clots are degraded and cleared from the circulation. The central enzyme in this pathway is the serine protease plasmin, which is formed by cleavage of the zymogen, plasminogen, at Arg_561_-Val_562_. The endogenous plasminogen activators are tissue plasminogen activator (tPA) and urokinase plasminogen activator (uPA). The structural homology of tPA and uPA is similar, however, they differ in their mechanism of action. Binding to fibrin enhances tPA-mediated plasminogen activation 1250-fold compared to the solution phase (1). In contrast, uPA can efficiently activate plasminogen in the absence of fibrin, either in solution or associated with its cellular receptor, urokinase plasminogen activator receptor (uPAR) (2). Plasmin sequentially cleaves fibrin into fibrin degradation products (FDPs). Measurement of the plasma level of fragment D-dimer, which consists of two D domains from adjacent fibrin monomers, is commonly used as marker of both ongoing thrombosis and fibrinolysis. Several inhibitors regulate fibrinolysis. The serpin, plasminogen activator inhibitor-1 (PAI-1), acts by rapidly forming a 1:1 complex to inhibit tPA and uPA (3, 4). Alpha2-antiplasmin (α2AP) is a fast-acting inhibitor of plasmin present at high circulating concentration (1 μM). Complexes of plasmin/α2AP (PAP) are cleared with a t1/2 of ~0.5 days and elevated levels are frequently used as a marker of pronounced fibrinolytic activity (5). Activated thrombin activatable fibrinolysis inhibitor (TAFIa) is a carboxypeptidase which exerts its anti-fibrinolytic function by cleaving c-terminal lysine residues from fibrin thereby downregulating the binding of tPA and plasminogen and subsequently reducing plasmin generation (6).

Dysregulation of the fibrinolytic system can lead to inefficient resolution of thrombi leading to thrombotic complications and indeed impaired endogenous fibrinolysis is an independent risk factor of cardiovascular events (7, 8). On the other hand, hyperfibrinolysis is associated with bleeding events such as trauma induced coagulopathy [Reviewed in (9)]. Current thrombolytic therapies carry an inherent risk of bleeding events and there is a pressing need to improve our understanding of the fibrinolytic system in order to tailor novel therapeutic strategies.

## Classical models of fibrinolysis

**Figure 1:**
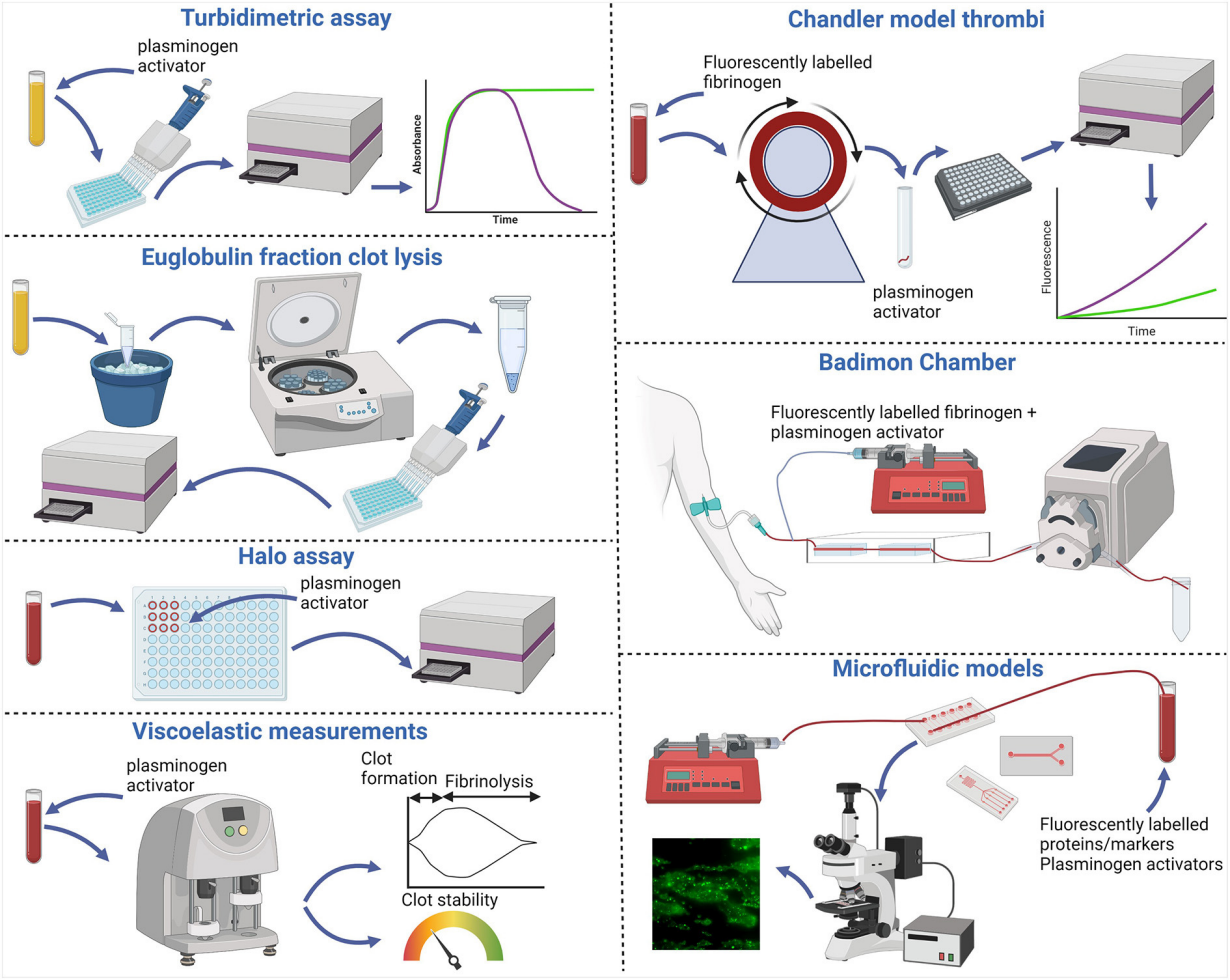
Fibrinolytic assays and models. Turbidimetric clot lysis assays involve clotting plasma in 96 well plates with the addition of calcium and thrombin in the presence of plasminogen activators. Change in absorbance is then measured using a spectrophotometer, increasing as clots form and decreasing during fibrinolysis. The Euglobulin fraction containing plasminogen, plasminogen activators and fibrinogen can be isolated by precipitation on ice cold acetic acid and collected *via* centrifugation. The pellet is then washed and solubilized before clotting with thrombin and the fibrinolytic activity then measured by change in absorbance. The Halo assay requires whole blood which is clotted with tissue factor in a “Halo” around the outside of a well of a 96 well plate. Plasminogen activators are then added and the absorbance increases as the clot lyses and is released into the well. Viscoelastic measurements are made in whole blood with added plasminogen activator using an analyzer. Data is outputted as traces [TEG^®^ (Haemonetics Corporation) and ROTEM^®^ (Werfen)] or a dial output [Quantra^®^ (Hemosonics LLC)]. Chandler model thrombi are formed after recalcifying whole blood or plasma with added fluorescently labeled fibrinogen and enclosing in tubing under rotation. Thrombi are then removed and bathed in plasminogen activator and samples are taken at regular intervals and change in fluorescence release is measured as a marker of fibrin degradation. The Badimon chamber uses non-anticoagulated blood taken directly from the donor. Plasminogen activators and fluorescently labeled fibrinogen can be mixed with the blood *ex vivo* which then flows over thrombogenic tissue within the specialized perfusion chambers at predefined shear stresses. Markers of fibrinolysis can be measured from collected effluent. Additionally, thrombi formed on the thrombogenic surface can be removed and fibrinolysis monitored as with the Chandler model. Whole blood microfluidic models use syringe pumps to either pull or push blood over microfluidic biochips. These can be commercially sourced or prepared in house and can have different channel sizes and conformations. Thrombus formation is initiated on thrombogenic coatings within the biochips and viewed *via* fluorescence microscopy. Various markers and fluorescently labeled proteins can be incorporated pre or post thrombus formation. Fibrinolysis can be monitored as change in fluorescence signal over time and effluents can be collected to monitor markers of fibrinolysis. Figure created with BioRender.com.

Classically, fibrinolysis was studied in static models, most commonly by measuring a change in absorbance over time which increases as the clot forms and decreases as it degrades (Figure 1). This technique can only be used with either platelet-poor or platelet-rich plasma, and not in whole blood due to interference of red blood cells. An adapted Euglobulin clot lysis time (ECLT) assay has been shown to be advantageous when investigating hyperfibrinolysis (Figure 1) (10). This assay uses the euglobulin fraction which allows for fibrinolytic activity to be detected with the endogenous tPA (10). The Halo assay is a plate-based assay where blood/plasma is plated in a ‘halo' around the periphery of the well (Figure 1) (11). This was developed to be a high-throughput thrombolysis assay using small sample volumes of citrated whole blood. Clotting is initiated using tissue factor and once the ‘halo' clot is formed it is overlayed with a plasminogen activator. The change in absorbance is monitored as the clot degrades and red blood cells are released over the center of the well. These assays are advantageous as they are easily accessible, adaptable and relatively high throughput as they are suited to a 96-well plate format. Clot lysis assays are also routinely performed on stored plasma material, rather than necessitating fresh whole blood. These static fibrinolytic assays are useful tools in understanding basic mechanisms of fibrinolysis but lack standardization resulting in inter-laboratory variation and a lack of healthy control ranges. Viscoelastic assays such as the TEG^®^ (Haemonetics Corporation), ROTEM^®^ (Werfen) and more recently the Quantra^®^ (Hemosonics LLC) allow for rapid measurement of clot strength and susceptibility to lysis in whole blood as point of care assays (Figure 1). TEG^®^ and ROTEM^®^ can both be run using either anticoagulated or non-anticoagulated blood which requires the samples to be processed immediately. These viscoelastic assays can detect hyperfibrinolysis but are insensitive to subtle changes in normal fibrinolytic activity. Optimized incorporation of tPA in these viscoelastic tests has allowed for rapid assessment of fibrinolysis in acute clinical settings such as trauma induced coagulopathy (12).

However, all of these assays discussed have the disadvantage that they do not consider the influence of shear stress on the structure of the fibrin network and the cellular composition and interactions. Furthermore, the dilutional effect and convectional removal of thrombolytics and fibrinolytic proteins is not replicated in the absence of flow.

The shear stress and the site of formation greatly influences the structure and composition of thrombi (13, 14). Thrombi formed under high shear are more concentrated in platelets than those that are formed under low shear which are more fibrin- and red blood cell-rich (13). However, within a thrombus, there is heterogeneity with areas that are rich in red blood cells ensnared in fibrin and those that are more complex consisting of packed platelets associated with von Willebrand factor, leukocytes and dense fibrin (15). Platelets are reported to account for 31% of thrombus volume in arterial thrombi obtained after thrombectomy whilst they account for only 0.4% in venous thrombi (16). Platelet-rich thrombi display enhanced resistance to lysis when compared to red blood cell-rich thrombi (17) which likely contributes to the failure of thrombolytic therapy in recanalizing some patients (18). Thrombi formed under shear stress tend to have a surface of tightly packed fibers that align in the direction of flow and an interior consisting of fibers that are more heterogenous and porous in nature (19, 20). These structural differences in turn affect the susceptibility to fibrinolytic degradation (21) with clots that consist of dense, tightly packed, thin fibrin networks being less susceptible (22, 23). Therefore, models incorporating physiological flow rates are advantageous and are increasingly utilized in studies of fibrinolysis to mimic the situation *in vivo* more accurately.

### Physiological flow models

#### Early flow models

**Figure 2:**
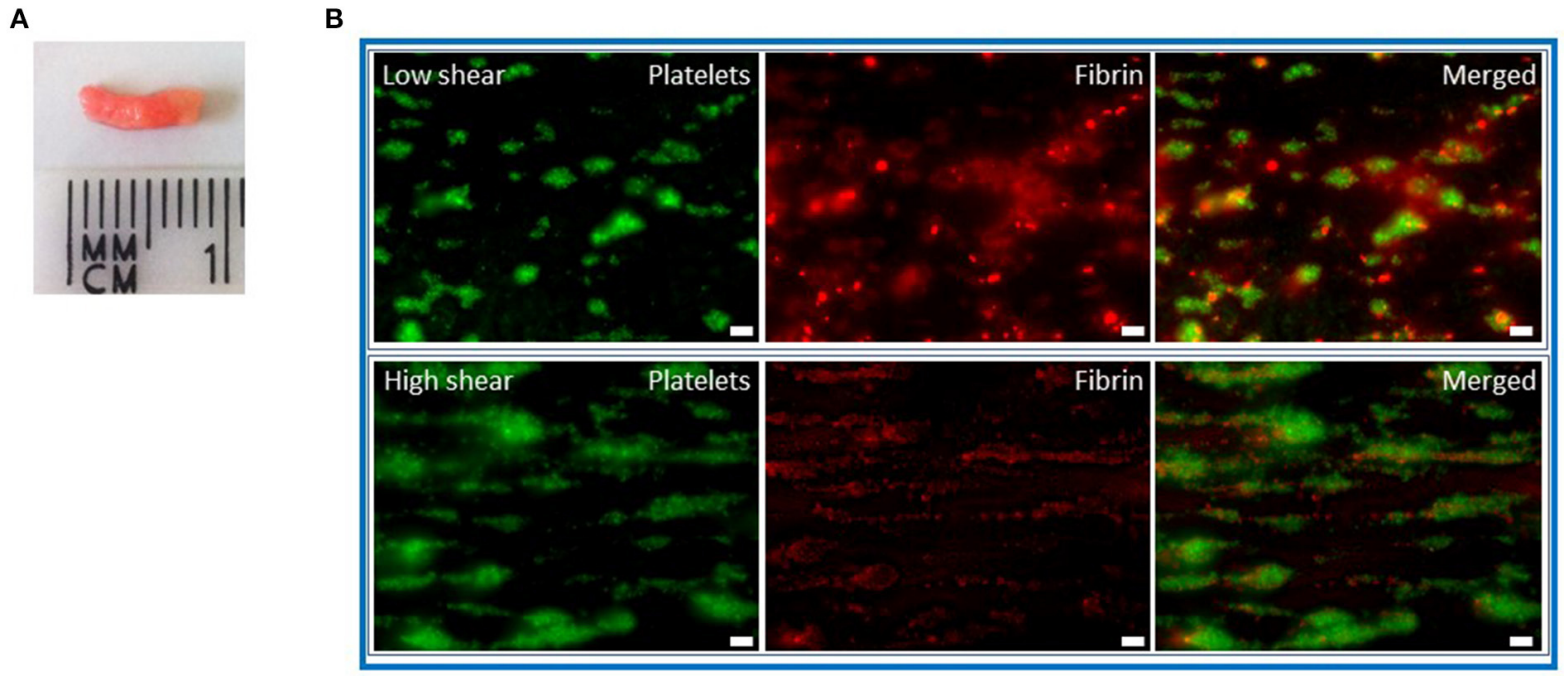
Whole blood thrombi under flow. **(A)** Chandler model thrombi formed from citrated whole blood that was recalcified (10.9 mM CaCl_2_). Thrombi were formed under rotation at a constant speed of 30 rpm for 90 min before being removed and washed in 0.9% saline. The platelet-rich white head is visible. **(B)** Recalcified whole blood thrombi were formed on a collagen (100 ng) and tissue factor (300 pM) surface in Cellix biochips. Thrombi formed under high shear (1,000 s^−1^) are more platelet-rich with less fibrin than those formed at low shear (250 s^−1^). DiOC6 (0.5 μg/ml) was included to label platelets (green) and fibrinogen-AF546 (75 μg/ml) added (red). Scale bars represent 10 μm.

The Chandler model was developed in the 1950s (24) and involves the formation of whole blood or plasma thrombi under flow (Figure 1). This simple but effective system forms thrombi in closed loops of polyvinyl tubing sealed using a short piece of tubing of a greater diameter and placed on a rotor at a constant speed. Whole blood or platelet-rich plasma thrombi formed under arterial shear rates mimic those found *in vivo* with a platelet dense head and fibrin rich tail (13) (Figure 2A). Incorporating fluorescently labeled fibrinogen during formation acts as a tracer to subsequently monitor release of FDPs. Allowing thrombi to proceed to full degradation confirms the sensitivity of this method in detecting differences in release of the degradation products (25). After thrombi have formed under flow, they can be lysed in a bathing solution alone, to monitor spontaneous lysis (26, 27), or with plasminogen activators (3, 27, 28). Release of fluorescent fibrin degradation products over time is directly proportional to the rate of lysis. Plasminogen activators at endogenous concentrations (29) can also be incorporated during thrombus formation under flow, however, the formation time must be decreased to account for simultaneous ongoing fibrinolysis (30). This system is also sensitive to detecting the influence of pharmacological agents on the susceptibility to lysis (31).

Activated Factor XIII (FXIIIa) crosslinks the fibrinolytic inhibitors TAFIa (32), PAI-2 (33) and α2AP (34) to fibrin. Using the Chandler model, we have demonstrated that it is crosslinking of α2AP that is primarily responsible for the antifibrinolytic function of FXIIIa (35, 36). Interestingly, we showed that the antifibrinolytic effect of FXIIIa cannot be visualized in static conditions, emphasizing the importance of incorporation of shear stress. This was subsequently shown by Rijken et al. in a compaction-based model of fibrinolysis (37). The Chandler model can be adapted to model physiological fibrinolytic challenges. For example manipulating the blood by hemodilution to model trauma induced coagulopathy permits us to visualize the efficacy of restoring fibrinogen, *via* supplementation with fibrinogen concentrates or cryoprecipitate, in stabilizing thrombi against degradation (38).

The Badimon Chamber uses *ex vivo* blood which is flowed over thrombogenic tissue, such as deendothelialized aorta or Type I collagen bundles (Figure 1) (39). This model has the unique feature that native venous blood is pumped through two cylindrical perfusion chambers with predefined shear stress arranged in series (40). The influence of antithrombotic therapy on thrombus formation can be monitored as thrombus size and release of D-dimer, as a marker of fibrinolysis (41, 42). The nature of this model also allows the direct study of endogenous tPA release from the endothelium after bradykinin infusion (29). Alternatively, fluorescently labeled fibrinogen and exogenous tPA can be administrated *via* a calibrated syringe-driver into the extracorporeal circuit. Fibrinolysis can be quantified as fluorescence release after removing the porcine aorta strips, with thrombi attached, and placing in tPA bathing fluid and/or as the concentration of fibrinolytic proteins quantified in effluents (29, 30).

### Microfluidic models of fibrinolysis

The use of microfluidic devices is a well-established technique for the study of thrombus formation under shear stress in normal and stenotic venous and arterial circulations. The experimental configuration varies greatly from one laboratory to another. The microfluidic device can be comprised of parallel-plate flow chambers (43) commercially available biochips or customizable biochips developed in-house using templates and molded polydimethylsiloxane (PDMS) plasma bonded (44, 45) or vacuum sealed (46) to glass slides or coverslips.

Coagulation is initiated as blood is flowed over surfaces coated with thrombogenic substances including tissue factor, collagen (most commonly of equine source), thrombin and von Willebrand factor and even hydrolyzed atheromatous material. The pattern of the coating varies from micro-spots to whole channel. Platelet-fibrin thrombi are visualized by staining platelets with lipophilic fluorescent dyes, most commonly 3,3′-dihexyloxacarbocyanine iodide (DiOC_6_), and incorporating fluorescently conjugated fibrinogen. The resulting 3D fibrin-platelet thrombi are visualized by fluorescence and brightfield microscopy with images acquired by a camera. Spatial and temporal analysis of changes in fluorescence can be quantified using imaging software.

The aforementioned flow models permit thrombus formation under flow conditions, yet fibrinolysis is primarily monitored under static conditions. We developed the first physiological microfluidic flow model of fibrinolysis to recapitulate the hemodynamic conditions under which fibrin is both formed and degraded (47). We adapted a well-established parallel-plate perfusion chamber (48) which is capable of forming thrombi with different hierarchal structures (49). Thrombi form with fibrin emanating from aggregated platelets surrounded by phosphatidylserine positive platelets (50). To tailor this model to the study of fibrinolysis we used a similar initial set up to the established model and perfused citrated whole blood (1,000 s^−1^) over a glass coverslip coated with micro-spots of fibrillar type I collagen in the presence or absence of recombinant human tissue factor. Platelets were labeled with DiOC_6_ or AlexaFluor labeled Annexin V to detect those that were phosphatidylserine positive and fluorescently labeled fibrinogen or plasminogen were added to the blood in the presence or absence of tPA or uPA. After 7 mins of thrombus formation a Hepes buffer, pH 7.45 was perfused. Fibrin degradation was measured as spatiotemporal changes in fluorescent fibrinogen signal. Dose-dependent fibrinolysis was observed in platelet-fibrin thrombi as change in fluorescence of fibrin(ogen). Fluorescently labeled plasma plasminogen primarily localized to platelet-associated fibrin(ogen) in the thrombus core (47). These findings are akin to an *in vivo* model examining plasminogen localization after laser injury (51). This model was sensitive to detecting differences in the susceptibility to lysis with a dose-dependent effect of tPA and uPA and demonstrated the balance of ongoing fibrin deposition and fibrinolysis. A higher molar concentration of uPA was required to observe lysis, consistent with the known fibrin specificity of tPA. Similarly, Loyau et al. (52) demonstrated the binding of both plasminogen and tPA in pre-formed platelet-fibrin thrombi and subsequent lysis using supraphysiological concentrations of the plasminogen activator in commercially available biochips (Vena8 Fluoro+ Biochips, Cellix Ltd.) (52). Thrombi formed in microfluidic devices display differences in platelet and fibrin deposition depending on the shear rates applied (Figure 2B). Fibrinolysis can be further quantified by measurements of plasmin activity, D-dimer, FDP and PAP in the effluents from these devices.

Microfluidic devices facilitate the modeling of surgical and pathophysiological conditions. Trauma induced coagulopathy was modeled by addition of exogenous tPA to mimic hyperfibrinolysis (53). This study used an eight-channel device which pulls the blood from a single outlet permitting simultaneous study of multiple conditions. These models are excellent tools for monitoring the influence of pharmacological manipulation of blood samples and have previously been used to monitor platelet deposition in response to P2Y1 and P2Y12 inhibitors and recombinant FVIII (54, 55). The experimental conditions can be manipulated to tease out the contribution of platelets/fibrin, for example using PPACK or hirudin to inhibit thrombin and subsequent fibrin formation. Inhibiting fibrin formation attenuates binding of both plasminogen (47) and tPA (52). Neutralization of the intrinsic pathway can be achieved by incorporation of corn trypsin inhibitor (CTI) (53). Importantly these devices can be used to study the efficacy of novel thrombolytic therapies. Using the Cellix Vena8 Fluoro^+^ chips, Huang et al. have demonstrated the platelet selectivity of fibrinogen-mimicking multi-arm nanovesicles as a vehicle for targeting thrombolytic therapy (56). This study combined experimental data generated in microfluidic devices with computational modeling further enhancing the power of this technique in developing our understanding of the dynamics of fibrinolysis and predicting kinetics of thrombolytic therapies (56).

Custom designed biochips allow for tailoring to specific experimental questions. An elegant study using PDMS chips bonded to a glass slide with an H-shape channel demonstrated the enhanced ability of tPA-conjugated colloidal micro-wheels in dissolving thrombi (44). The unique chip design represents vascular and extravascular compartments. The tPA micro-wheels were perfused into one vertical channel and, after applying a magnetic field to induce a corkscrew motion, they were visualized to rapidly promote fibrin dissolution in the horizontal “injury channel” (44). This method of combining mechanical and bulk dissolution has been further refined to incorporate plasminogen thereby overcoming rate limiting depletion of this zymogen (57). Although a constant flow rate is applied, the occlusion of the vessel wall leads to increasing shear stress at the site of thrombus formation (58). However, *in vivo*, blood can be diverted into other branches therefore releasing pressure. This effect is not recapitulated in single channel microfluidics leading to the development of bifurcating biochips which are designed to allow for diversion of blood (46, 58). In a microfluidic model of sterile thrombus occlusion, high interstitial hemodynamic forces promote the formation of neutrophil extracellular traps (NETs) (59). NETs are formed from expulsion of the nuclear and cytoplasmic contents from activated neutrophils resulting in extrusions consisting of chromatin adorned with DNA and proteins. NETs have been shown to exert antifibrinolytic effects (60–62). In this sophisticated model, NETosis was triggered under arterial shear conditions, yet was largely absent under venous conditions (59). Fibrin was observed to repress the formation of NETs while addition of exogenous tPA under arterial shear rates facilitated NET formation (63).

### Point of care devices

There is a distinct requirement for rapid and consistent measurements of thrombus formation and thrombolysis. Balancing this with incorporating the impact of shear on thrombus formation is a challenge for the field. Recently there have been a number of point-of-care (POC) devices that aim to tackle this. The Global Thrombosis Test (GTT) (Thromboquest Ltd., UK) uses native non-anticoagulated blood to form thrombi under high shear with blood passing through a ball bearing situated within the chamber. This POC device has recently highlighted an association between clot architecture and platelet reactivity with endogenous fibrinolysis in patients suffering a STEMI (64, 65). Impaired endogenous fibrinolysis has now been proposed as a marker of cardiovascular risk (66, 67).

The Total-Thrombus-formation Analysis System (T-TAS) (Fujimori Kogyo Co. Ltd., Japan) is an integrated automated microchip flow chamber system designed to monitor thrombus formation. This system does not offer customization, as it uses chips with predefined shear stresses and procoagulant precoated surfaces. Nonetheless, the T-TAS analyzer has demonstrated the efficacy of tPA added to blood prior to thrombus formation at high and low shear. Inhibition of PAI-1 by the small molecule inhibitor (PAI-039) had a moderate effect in enhancing the fibrinolytic potential of tPA in thrombi formed in the T-TAS under arterial shear rates (68).

### Limitations of flow models

Whilst flow models can recapitulate physiological dynamics of thrombus formation and can be used for screening of novel antithrombotic or thrombolytic therapies, they of course do not consider ongoing pharmacodynamics that may happen *in vivo*. For whole blood flow models there is a requirement for freshly obtained anticoagulated blood samples that should be used within 4 h of sampling but not within the first 15 to 20 mins (69). This puts a time restriction on the experimental conditions that can be explored and is particularly of note when studying fibrinolysis, as time for both thrombus formation and degradation must be accounted for. The Global thrombosis test uses non-anticoagulated blood and therefore must be processed immediately.

Assays such as the Chandler model thrombus system allow for comparison of multiple conditions simultaneously and generate a thrombus with structural features that resemble *in vivo* thrombi. However, this is a closed system and therefore does not mimic removal of components as occurs in the circulation and monitoring of the fibrinolytic arm is achieved under static conditions which is sub-optimal. POC devices provide ease of use and standardized methods of obtaining quantitative data which are attractive for clinical assessment of fibrinolytic potential. However, their lack of adaptability does not lend itself to mechanistic studies.

Visualization of thrombus formation and resolution in microfluidic devices requires fluorescence microscopes and cameras capable of appropriate acquisition times and magnifications. The purchase of commercial biochips and associated pumps mean that these models can be very costly, particularly during initial set-up. In-house preparation of biochips using PDMS can offer more affordable and adaptable biochips, but this requires the production of the initial mold, which can be costly, and necessitates design expertise. These assays are difficult to standardize due to the vast range of variables including types of biochips, coating substances, channel conformation and methods of detection. There has been a drive by the International Society on Thrombosis and Haemostasis Scientific Standardization Committee on Biorheology to standardize models of thrombus formation and platelet function in flow-based assays (69). These recommendations are applicable to fibrinolysis models, alongside additional complexities to consider including choice of plasminogen activator, its concentrations and whether it is included pre- or post-thrombus formation in addition to the various methods of monitoring fibrinolysis. Of course, donor variability further confounds these studies which leads to differences in platelet response and surface coverage (70), inter-individual variations in fibrin structure and deposition of hemostatic proteins.

The microvasculature expresses the adhesive glycoprotein thrombomodulin (TM) which drives the anticoagulant function of the endothelium by facilitating the activation of the anticoagulant, protein C. However, TM also participates indirectly in fibrinolysis by promoting thrombin-mediated activation of TAFI. Vascular endothelial cell expression of these fibrinolytic factors, including tPA, uPA is altered in response to shear stress (71, 72). PAI-1 is secreted from endothelial cells (73) and is upregulated by dysfunction of the endothelium due to elevated proinflammatory cytokines (74, 75) and C-reactive protein (76, 77). Endothelial regulation of fibrinolysis is an important component in dictating the overall fibrinolytic response within the vessel wall. Therefore, there is a current drive to develop endothelialized models that can more accurately recapitulate the vasculature not only in shear stress but also contribution of the endothelium.

## Conclusions

The thrombus architecture is a dynamic environment and the shear forces that thrombi are exposed to shape the resulting fibrin network and cellular composition. Models that incorporate hemodynamic forces are an excellent tool to unpick the molecular and cellular interactions that dictate thrombus formation, structure, stability and resistance to lysis. The ability to use human blood *ex vivo* under physiological flow rates mitigates some of the issues of species differences experienced when using animal thrombosis models. Furthermore, this is in line with initiatives to reduce or replace the use of animals in scientific research such as that of the National Center for Replacement, Refinement and Reduction of animals in research. There have been considerable advancements in microfluidic models within the last decade to monitor fibrinolysis, a tool that has been seriously lacking thereby prohibiting our understanding of this complex system. These novel models offer adaptability to mimic shear rates experienced in different anatomical locations and vascular beds and provide important mechanistic insights into the regulation of thrombus stability. Furthermore, they are an excellent tool to facilitate the development and testing of novel thrombolytic therapies and/or drugs that may influence the responsiveness of thrombi to antithrombotic drugs.

## Author contributions

CSW and NJM conceived the ideas, researched, and wrote and edited the manuscript. All authors contributed to the article and approved the submitted version.

## Funding

CSW and NJM are supported by the British Heart Foundation (PG/20/17/35050) and a NC3Rs-British Heart Foundation Studentship (NC/W001810/1).

## Conflict of interest

The authors declare that the research was conducted in the absence of any commercial or financial relationships that could be construed as a potential conflict of interest.

## Publisher's note

All claims expressed in this article are solely those of the authors and do not necessarily represent those of their affiliated organizations, or those of the publisher, the editors and the reviewers. Any product that may be evaluated in this article, or claim that may be made by its manufacturer, is not guaranteed or endorsed by the publisher.
